# Decentralized Navigation with Optimality for Multiple Holonomic Agents in Simply Connected Workspaces

**DOI:** 10.3390/s24103134

**Published:** 2024-05-15

**Authors:** Dimitrios Kotsinis, Charalampos P. Bechlioulis

**Affiliations:** 1 Division of Systems and Automatic Control, Department of Electrical and Computer Engineering, University of Patras, Rio, 26504 Patras, Greece; up1059482@ac.upatras.gr; 2 Athena Research Center, Robotics Institute, Artemidos 6 & Epidavrou, 15125 Maroussi, Greece

**Keywords:** multi-agent systems, motion planning, decentralized navigation, navigation function, optimal motion planning, multi-agent Poli-RRT*

## Abstract

Multi-agent systems are utilized more often in the research community and industry, as they can complete tasks faster and more efficiently than single-agent systems. Therefore, in this paper, we are going to present an optimal approach to the multi-agent navigation problem in simply connected workspaces. The task involves each agent reaching its destination starting from an initial position and following an optimal collision-free trajectory. To achieve this, we design a decentralized control protocol, defined by a navigation function, where each agent is equipped with a navigation controller that resolves imminent safety conflicts with the others, as well as the workspace boundary, without requesting knowledge about the goal position of the other agents. Our approach is rendered sub-optimal, since each agent owns a predetermined optimal policy calculated by a novel off-policy iterative method. We use this method because the computational complexity of learning-based methods needed to calculate the global optimal solution becomes unrealistic as the number of agents increases. To achieve our goal, we examine how much the yielded sub-optimal trajectory deviates from the optimal one and how much time the multi-agent system needs to accomplish its task as we increase the number of agents. Finally, we compare our method results with a discrete centralized policy method, also known as a Multi-Agent Poli-RRT* algorithm, to demonstrate the validity of our method when it is attached to other research algorithms.

## 1. Introduction

Motion Planning (MP) is one of the fundamental problems in robotics and a great amount of control algorithms have been proposed to solve it. The main purpose is to find methods to discover safe and, to the greatest extent possible, optimal trajectories either for a single mobile robot/agent system (Single-Agent System—SAS) or a multiple mobile robots/agents system (Multi-Agent System—MAS), which navigate the robot/agents’ journey from any initial point of the workspace to their destination. In the research community and industry, controlling MASs is a state-of-the-art task, as these systems can complete tasks faster and more efficiently than SAS. Despite their effectiveness, developing control policies that result in suitable trajectories with minimum cost (in terms of the agent’s path distance and input energy) is extremely challenging.

There are two control policies used to navigate a MAS: the centralized policy (CP), in which the agents are treated as a whole system and each agent is guided by one global controller, and the decentralized policy (DP), where each agent has its own control policy and exchanges information with the others to reach its destination safely without collisions. Designing DP controllers is more challenging than the centralized one, as the CP simplifies the problem and the agents have all the information about the environment and the goal position of all agents. On the other hand, in DP, the constraint of agents’ communication plays a vital role in the control design (for more information about the differences between CP and DP, the reader is referred to [1]). There is an abundance of studies that utilize these policies to efficiently navigate the agents, helping them to accomplish their tasks. They are mostly published in the field of exploration [2,3,4,5,6,7] and formation [8,9,10,11,12,13].

Furthermore, numerous researchers have proposed methods for navigating mobile robots to a final position; see [14,15,16,17,18]. Some of these studies use a non-degenerate navigation function [19], creating a negated gradient potential field that is attractive towards the goal configuration and repulsive regarding obstacles and the workspace boundary. The first studies to apply the above function to help mobile agents navigate to their destination without facing any conflicts are [20,21] which describe a CP and DP, respectively. We have to mention that this type of controller does not provide strict global navigation, because a vector field in any manifold that has a unique attractive point should have at least as many saddles as obstacles [19]. Related multi-agent navigation function methods can be found in [22,23,24,25,26,27].

As we mentioned earlier, the ultimate goal for MAS navigation is to design methods that can find the optimal path for the agents to follow. In SAS, there are discrete (DM) and continuous methods (CM) that accomplish this task. In DM, there is an RRT* algorithm with various versions [28,29]; this algorithm modifies the robot’s path in order to decrease the minimum path length. The CM relies on Reinforcement Learning (RL)-based methods that use iterative algorithms to converge in an optimal navigation policy [30,31,32]. It is common in MAS to use methods based on Deep Reinforcement Learning (DRL), which is a powerful tool that combines neural networks and RL algorithms that allow each agent to learn from its interactions with the environment [33,34,35,36,37,38]. Despite the effectiveness of the RL-based methods, the main disadvantage in MAS is the computational complexity and abundance of data required to converge to the global policy.

Therefore in this paper, we are going to introduce a sub-optimal approach for navigating a MAS in planar simply connected workspaces (i.e., workspaces without any internal obstacles). We refer to them as sub-optimal because we begin by finding the optimal navigation for each agent separately with a novel off-policy iterative method [30] that is used to prevent the computational complexity of global MAS and, subsequently, we design a DP controller for multi-agent navigation, where we use the negated gradient of a navigation function from [19]. We apply a DP method to our MAS problem, as every agent should operate independently from the others. This is due to the fact that each agent possesses predetermined information about its optimal navigation and follows this policy to its destination when it is isolated from the others. Additionally, we adopt the approach from [21,26], referred to above, to design the controller for each agent in order to prevent collisions, and this approach also demands fewer numerical operations than the CP of [20], thus relaxing the requirements of the solution. Hence, the main contributions of this work are summarized as follows:

- We navigate each agent with a sub-optimal policy to its destination. To the best of our knowledge, this is the first work based on artificial potential fields that introduces optimality within a multi-agent navigation framework.
- No collision with other nearby agents or the workspace boundary occurs.
- Knowledge about the current position of the nearby agents and not their destination is required.
- The complexity is rendered linear with respect to the number of the agents and, if combined with the recent work [39], may be fixed.

Additionally, to demonstrate the efficiency of the proposed controller, in our simulation study, we examine how much the sub-optimal trajectory of each agent deviates from the optimal one of the SAS, how much time the overall system needs to accomplish its task, and how the time taken changes as we increase the number of agents starting simultaneously from their initial position. Also, we consider the results obtained using the Multi-Agent Poli-RRT* method, which was proposed by [40], in order to compare the validity of our method.

The rest of the paper is organized as follows: Section 2 formulates the problem. Section 3 describes the proposed decentralized navigation policy. Section 4 provides the proof of correctness of our approach. Section 5 presents the simulation results. Finally, Section 6 concludes the work and proposes future research directions.

## 2. Problem Formulation

Let us assume that we have a sphere manifold (ball set) $B_{r_{0}}\left(q\right)=\{x\in R^{2}:||x-q||<r_{0}\}$. Inside this set, we define a *simply connected workspace* $W\subset B_{r_{0}}\left(q\right)$ and its boundary, which is given by ∂W. There are *m mobile agents* (or *robots*) in *W*, with each one of them occupying a disk $A_{i}=\{q\in W:||q-q_{i}\left|\right|\leq r_{i}\}$, i=1,…,m where $q_{i}$, $r_{i}$ denote the center and radius of the disk, respectively. The *configuration space* is spanned by $q={\left[q_{1}^{T},\ldots ,q_{m}^{T}\right]}^{T}$. The *initial position* vector is defined as $\bar{q}={\left[{\bar{q_{1}}}^{T},\ldots ,{\bar{q_{m}}}^{T}\right]}^{T}$ and the *desired destination* vector as $q_{d}={\left[q_{d1}^{T},\ldots ,q_{dm}^{T}\right]}^{T}$. The motion of each agent follows a single integrator model:

(1) $$\begin{array}{c} {\dot{q}}_{i}=u_{i},\;i=1,\ldots ,m \end{array}$$

where $u_{i}$ denotes the velocity command applied to each agent. In particular, $u_{i}$ will be a sub-optimal decentralized control policy which will navigate the agent to its destination and prevent any conflicts/collisions with other agents and the workspace boundary ∂W. Also, the controller possesses knowledge about the current position of other nearby agents, but not their destination. Thus, every agent sees the others as moving obstacles in *W*.

Moreover, we assume that the workspace is spacious, and at every point of it, each agent is capable of avoiding any conflict, since in this paper, we mainly focus on the functionality and the appropriate design of the sub-optimal control policy. Thus, we exclude narrowly connected workspaces, where the agents need global coordination to navigate towards their destination to alleviate blocking issues, such as in [41]. We are going to deal with this issue in future work. Finally, we define the following infinite-horizon cost function for each agent:

(2) $$\begin{array}{c} V_{u_{i}}\left(\bar{q_{i}}\right)=\int_{0}^{\infty }\left[Q\left(q_{i}\left(\tau ;\bar{q_{i}}\right);q_{di}\right)+R\left(u_{i}\left(\tau ;\bar{q_{i}}\right)\right)\right]d\tau \end{array}$$

where $\bar{q_{i}}$ is the starting point of the agent in *W*. Additionally, we define the *state-related cost term Q* and the *input-related cost term R*, respectively:

(3) $$\begin{array}{c} Q\left(q_{i};q_{di}\right)=w||q_{i}-q_{d}i{\left|\right|}^{2} \end{array}$$

(4) $$\begin{array}{c} R\left(u_{i}\right)=\left(1-w\right)\left|\right|u_{i}{\left|\right|}^{2} \end{array}$$

where w∈(0,1) is a weight parameter and ||.|| denotes the Euclidean norm. The metric (2) along with (3), (4) form a classical function from Optimal Regulation theory [42]. The state-related term (3) expresses the minimization of the settling time of the system (1) and the input-related term (4) penalizes the control input’s Euclidean norm, which allows us to calculate the energy expenditure of the system (1). Furthermore, the weight’s value plays a vital role in the final result of the metric (2). It depends on what specification (reducing settling time or input energy) we desire to pass on to our system.

In summation, the aforementioned metric examines two factors: the first one is the amount of time that the whole MAS needs to reach the goal configuration and the second one is the energy of the control input signal, which is spent by each agent in order to successfully navigate the workspace. Thus, the objective of this work is to design appropriate velocity control policies $u_{i}$ such that collisions among the agents and the workspace boundary are safely avoided and the overall cost

$$V_{MAS}=\sum_{i=1,\ldots ,m}V_{u_{i}}\left(\bar{q_{i}}\right)$$

is rendered as small as possible for any $\bar{q_{i}}\in W$, i=1,…,m.

## 3. Decentralized Navigation

In this section, we briefly discuss the sub-optimal DP. First, we are going to mention the definition of the navigation function, its properties, and the sub-optimal decentralized control law that each agent is equipped with. The control law for each agent, as mentioned before, navigates them to their destination optimally with respect to (2) and prevents them from colliding with the workspace boundary. Hence, we are going to briefly present the off-policy RL-based method of [30], which we use to find the independent optimal navigation policy of each agent and determine the function that provides a warning regarding conflicts between the agents and suggestions of how to resolve such issues.

### 3.1. Navigation Function

According to [19], a Navigation Function (NF) is a real-valued map defined through cost functions, whose negated gradient field is attractive towards the goal configuration and repulsive with respect to obstacles. In the aforementioned paper, it has been shown that it is not possible to achieve strict global navigation, (i.e., with a globally attracting equilibrium state), because a smooth vector field on any sphere world, which has a unique attractor, should have at least as many saddles as obstacles. Our assumption that we have spherical robots (and thus spherical obstacles) does not constrain the generality of this work, since it has been proven [19] that navigation properties are invariant under diffeomorphisms. A NF can be defined as follows:

**Definition 1**  ([19])**.** *Let $F\subset R^{n}$ be a compact connected analytic manifold with a boundary. A map φ:F→[0,1] is a navigation function if*

*1.*  *It is analytic on F;*
*2.*  *It has only one minimum at $q_{d}\in int\left(F\right)$;*
*3.*  *Its Hessian at all critical points (zero gradient vector field) is full rank;*
*4.*  *$\lim_{q\to \partial F}\varphi \left(q\right)=1$.*

Since we design controllers with the DP, where each agent needs to know the current position of the other close-distance agents with the goal of preventing collisions, and each of them operates solely based on its own navigation law, we consider the above class of navigation functions from [26] for each agent:

(5) $$\begin{array}{c} \varphi_{i}=\sigma_{d}\circ \sigma \circ \hat{\varphi_{i}}={\left(\frac{\gamma_{i}}{\gamma_{i}+G_{i}}\right)}^{\frac{1}{k}} \end{array}$$

which is a composition of $\sigma_{d}\left(x\right)=x^{\frac{1}{k}}$, $\sigma \left(x\right)=\frac{x}{1+x}$ and

(6) $$\begin{array}{c} \hat{\varphi_{i}}=\frac{\gamma_{i}}{G_{i}} \end{array}$$

is a polar function, where the column vector $\hat{\varphi }={\left[\hat{\varphi_{1}},\ldots ,\hat{\varphi_{m}}\right]}^{T}$ has its minimum at $q_{d}\in int\left(F\right)$, *k* is a positive constant and $\gamma_{i}^{-1}\left(0\right)$ denotes the desirable set and $G_{i}^{-1}\left(0\right)$ the set to avoid. We choose $\gamma_{i}={\gamma_{di}}^{k}$ where $\gamma_{di}=V_{u^{*}}^{\left(i\right)}$. This function is equal to the optimal cost function, which will be calculated with a novel iterative method such as the one in [30], which will be described in the next section. Thus, the gradient of $V_{u^{*}}^{\left(i\right)}$ shows the optimal navigation of every agent towards its desired destination. Notice that the cost function $V_{u^{*}}^{\left(i\right)}$, as a proper Lyapunov function, possesses the following properties [20]: (1) it is non-negative, (2) $V_{u^{*}}^{\left(i\right)}\left(q\right)=0\Leftrightarrow q=q_{di}$, (3) it is differentiable with regard to its variables, and (4) its Hessian is positive-definite. Hence, the sub-optimal control law for each agent will be given by

(7) $$\begin{array}{c} u_{i}=-u_{si}\frac{\nabla \varphi_{i}}{||\nabla \varphi_{i}\left|\right|+\epsilon } \end{array}$$

with $u_{si}=\sqrt{\frac{w}{1-w}}\left|\right|q_{i}-q_{di}\left|\right|$ as discussed in [30], which leads the agent smoothly to its destination while avoiding collisions ($u_{i}$ is co-linear with $\nabla \varphi_{i}$) and ϵ>0 is a small constant.

**Remark 1.**  *The variable ϵ takes proximate values between $10^{-4}$ and $10^{-8}$ to avoid the singularity at the desired configuration. We choose the value according to the system’s dynamic.*

**Remark 2.**  *The estimation of the cost function (2) affects the behavior of the decentralized navigation controller (7). We can see this easily from the gradient of NF, which is equal to*

(8) $$\begin{array}{c} \nabla \varphi_{i}\left(q_{i}\right)=\frac{1}{{\left({\gamma_{di}}^{k}+G_{i}\right)}^{\frac{2}{k}}}\left({\left({\gamma_{di}}^{k}+G_{i}\right)}^{\frac{1}{k}}\nabla \gamma_{di}-\gamma_{di}\nabla {\left({\gamma_{di}}^{k}+G_{i}\right)}^{\frac{1}{k}}\right) \end{array}$$

where $\gamma_{di}$ is equal to the estimated function $V_{u^{*}}^{\left(i\right)}$. As we observe in (8), the value and gradient of the $\gamma_{di}$ for any $q_{i}\in W$ affect the optimal reactive navigation of each agent and the way in which the agents interact to prevent conflicts among them.

### 3.2. Individual Optimal Policy

As we explained in the previous section, the control law for each agent involves an optimal navigation policy that leads it to its destination with minimal cost (2). Specifically, the function $\gamma_{di}=V_{u^{*}}^{\left(i\right)}$ is the estimation of the optimal cost function extracted by an off-policy iterative method presented in [30]. In the present work, such an optimal policy is considered to be known and in this section, we are going to briefly analyze how to extract it for a simply connected workspace. More specifically, we adopt the method described in [30] to calculate optimal policies based on which the authors propose an off-policy iterative RL-based method that identifies control laws by minimizing a certain infinite-horizon cost function. The authors of the study chose the off-policy approach [43] because it has a reduced computational cost compared to the on-policy. Their method starts with an initial admissible control policy which leads the system to its goal for any starting point within *W*. In practice, they define the initial policy with an Artificial Harmonic Potential Field (AHPF)-based method [44]. Moreover, they apply the Zeroing Barrier Function theory [45] to converge to the optimal control policy and also keep the agent at a safe distance from the workspace boundary ∂W. The approximation of the optimal cost function $V_{\hat{u}}^{\left(i\right)}$ is conducted by a Gaussian Radial Basis Functions (RBFs) Neural Network [46], whereas its gradient reveals the optimal navigation velocity of the agent.

**Figure 1:**
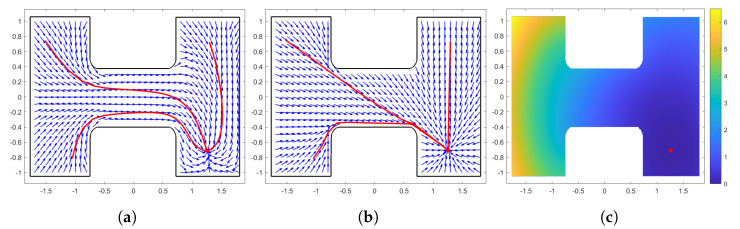
(**a**) Initial Policy. (**b**) Optimal Policy. (**c**) Optimal Cost Value.

In Figure 1, we show a simple example of the aforementioned algorithm. In this example, a point robot is in a simply connected workspace and we want to find its optimal navigation policy for any starting point in *W*. Figure 1a shows the initial admissible policy designed by the AHPF-based method and Figure 1b the optimal policy in which the algorithm converges. We observe in these two figures that the robot traverses the shortest distance with the optimal policy according to the three starting points. Finally, Figure 1c presents the minimum value of the cost function (2). As we can see, the algorithm achieves a sufficient approximation of the optimal solution within the workspace *W*. Nevertheless, when multiple agents navigate within the same workspace, collisions may occur, which will be handled by the following repulsive terms relating to nearby agents at risk of imminent collisions.

### 3.3. Resolving Conflicts via the Terms $G_{i}$

In this section, we will refer to mathematical tools and terminology from [20,26] in order to define the function $G_{i}$ that is in charge of resolving the conflicts that lead to collisions among the agents. More specifically, the function $G_{i}$ is different for each agent and shows whether the agents are close enough to collide with each other or with the workspace boundary. Hence, we can define this function as described below:

(9) $$\begin{array}{c} G_{i}=G_{bi}G_{ai} \end{array}$$

where $G_{bi}$ is the function of the *i*-th agent for avoiding conflicts with the boundary and $G_{ai}$ with the other agents. When one of these two functions approaches zero value, it means that the agent will face a conflict with some other nearby agents or the workspace boundary ∂W.

#### 3.3.1. Calculate Function $G_{bi}$

The function $G_{bi}$ is selected as

(10) $$\begin{array}{c} G_{bi}=b_{wi} \end{array}$$

where $b_{wi}$ is a distance metric between the agent and the workspace boundary. In [20,26] $b_{wi}$ defines the metric distance from the center of *W* to the agent’s center, because a spherical world is assumed. Since we deal with generic simply connected workspaces, we cannot use this metric. Therefore, we define its equivalent as follows:

(11) $$\begin{array}{c} b_{wi}=d^{2}\left(q_{i}\right)-r_{i}^{2} \end{array}$$

where $d\left(q_{i}\right)=\min_{z\in \partial W}\left|\right|q_{i}-z\left|\right|$.

#### 3.3.2. Calculate Function $G_{ai}$

Before defining the function $G_{ai}$, we need to refer to some mathematical tools and definitions and assume that each agent tracks other nearby agents as moving obstacles. So *A* will symbolize the examining agent and $O_{i},\;i=1,\ldots m-1$ will be the set of moving obstacles of *A*. The robot proximity function, which is a measure for calculating the distance between the agent and the obstacles, is defined as follows:

(12) $$\begin{array}{c} \beta_{A,i}={\left(q_{A}-q_{i}\right)}^{2}-{\left(r_{A}+r_{i}\right)}^{2} \end{array}$$

We will use the term *relation* to describe an imminent collision between one agent and a moving obstacle in *W*. A *binary relation* is a relation between the agent and a single obstacle. Any relation can be expressed as a set of binary relations. Finally, the *relation level* is the number of elements in the set $O_{i},\;i=1,\ldots m-1$.

**Figure 2:**
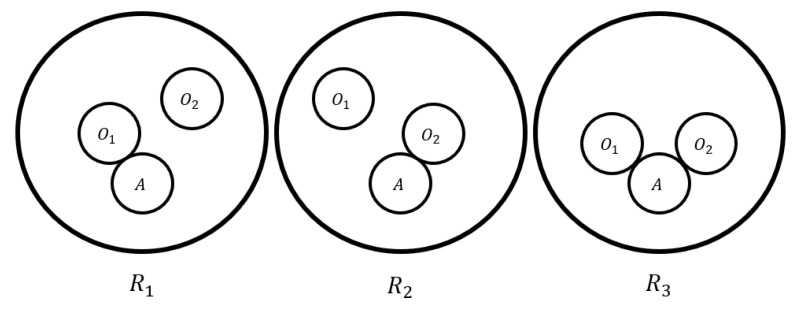
The relations which an agent has when a workspace involves three mobile agents.

Using the aforementioned terminology, we can define the set which involves the relations in level-*l* as $R^{\left(l\right)}=\left\{R_{i}^{\left(l\right)}:i=1,\ldots ,s_{l}\right\}$ where sl=(m−1l) and $R_{i}^{\left(l\right)}$ is the *i*-th set of binary relations in level-*l*. In the same manner, we define the complementary set of $R_{i}^{\left(l\right)}$, which is $\bar{R_{i}^{\left(l\right)}}=\left\{R_{j}^{\left(l\right)}:i\neq j\cap j=1,\ldots ,s_{l}\right\}$. To keep things simple, we use $R_{i}$ and $\bar{R_{i}}$, with i=1,…,S and $S=\sum_{l=1}^{m-1}s_{l}$, to refer to the relation and their complementary relation, respectively. For example, in Figure 2, we see the relations of an agent which is in a workspace with two other mobile obstacles. The total relations are S=3 and the maximum level of relations is two. For every relation shown in Figure 2, we have the following: on the left side the set $R_{1}=\left\{\left(A,O_{1}\right)\right\}$ and $\bar{R_{1}}=\left\{\left(A,O_{2}\right)\right\}$, on the center $R_{2}=\left\{\left(A,O_{2}\right)\right\}$ and $\bar{R_{2}}=\left\{\left(A,O_{1}\right)\right\}$ and finally, on the right $R_{3}=\left\{\left(A,O_{1}\right),\left(A,O_{2}\right)\right\}$ and $\bar{R_{3}}=\emptyset$.

A *Relation Proximity Function (RPF)* is a measure of the distance between the agent and the obstacles involved in a relation, and each relation has its own RPF. It is equal to

(13) $$\begin{array}{c} b_{R_{i}}=\sum_{j\in R_{i}}\beta_{j} \end{array}$$

where $\beta_{j}$ is the proximity function (12) with *j* denoting each binary relation of the set $R_{i}$. The property of RPF is that when this function has zero value, it shows that the agent conflicts with one or multiple obstacles according to the level of relation. A *Relation Verification Function (RVF)* is defined by

(14) $$\begin{array}{c} g_{R_{i}}\left(b_{R_{i}},B_{\bar{R_{i}}}\right)=\left\{\begin{array}{cc} b_{R_{i}}+\lambda \frac{b_{R_{i}}}{b_{R_{i}}+{\left(B_{\bar{R_{i}}}\right)}^{1/h}} & \mathrm{if}\;i\neq S\\ b_{R_{i}} & \mathrm{if}\;i=S \end{array}\right. \end{array}$$

where λ,h>0 and $B_{\bar{R_{i}}}=\prod_{k\in \bar{R_{i}}}b_{k}$. The RVF is zero if a relation holds while no other relation from the same level holds, and has the following properties: (a) $\lim_{x\to 0}\lim_{y\to 0}g_{R}\left(x,y\right)=\lambda$ and (b) $\lim_{y\to 0}\lim_{x\to 0}g_{R}\left(x,y\right)=0$.

**Figure 3:**
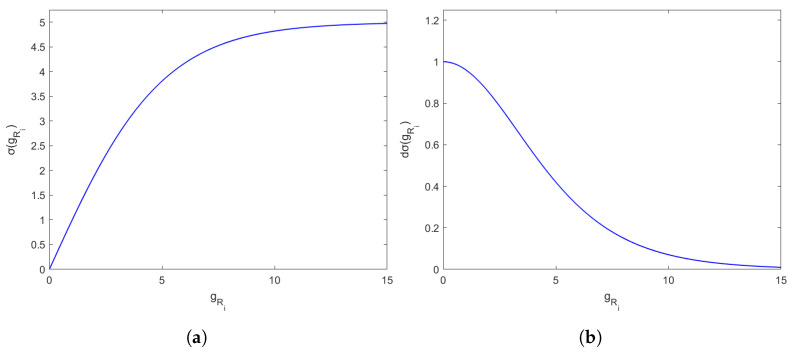
(**a**) Sigmoid function and (**b**) gradient of sigmoid function for $\tau_{m}=5$.

It should be noted that due to the fact that we assume we are working with large workspaces, the function $g_{R_{i}}$ can take large values in some relation $R_{i}$, affecting the numerical stability of the algorithm. Consequently, we add a sigmoidal function, as defined below, which ensures the values are adequately small. Figure 3 shows the graph of the sigmoidal function along with its gradient:

(15) $$\begin{array}{c} \sigma_{\tau_{m}}\left(g_{R_{i}}\right)=\tau_{m}\frac{e^{g_{R_{i}}/\tau_{m}}-e^{-g_{R_{i}}/\tau_{m}}}{e^{g_{R_{i}}/\tau_{m}}+e^{-g_{R_{i}}/\tau_{m}}} \end{array}$$

with $\tau_{m}=5$.

Having defined the RVF, then we can calculate the term $G_{ai}$ as follows:

(16) $$\begin{array}{c} G_{ai}=\prod_{j=1}^{S}\sigma_{\tau_{m}}\left(g_{R_{j}}\right) \end{array}$$

with its gradient given by:

(17) $$\begin{array}{c} \nabla G_{ai}=\sum_{j=1}^{S}\left(\prod_{k=1,j\neq k}^{S}\sigma_{\tau_{m}}\left(g_{R_{k}}\right)\right)\sigma_{\tau_{m}}^{\prime }\left(g_{R_{j}}\right)\nabla g_{R_{j}} \end{array}$$

In Equation (17) we see another benefit of the sigmoid function. When a relation provides us with a big value from its RVF, the derivative of the sigmoidal function eliminates the gradient of the relation’s RVF. As a result, when an agent is quite away from the others, it is navigated toward its destination by the optimal policy.

## 4. Proof of Correctness

Let ϵ>0. Define a ball $B_{j,l}^{\left(i\right)}\left(\epsilon \right)=\left\{q:0<g_{R_{j}^{\left(l\right)}}^{\left(i\right)}\left(q\right)<\epsilon \right\}$. According to [20], we discriminate the following topologies for the function $\phi_{i}$:

1. The destination point $q_{di}$.
2. The free space boundary: $\partial F\left(q\right)=G_{i}^{-1}\left(\delta \right),\delta \to 0$.
3. The set near collisions: $F_{0}\left(\epsilon \right)={⋃}_{l=1}^{m-1}{⋃}_{i=1}^{s_{l}}B_{j,l}^{\left(i\right)}\left(\epsilon \right)-\left\{q_{d_{i}}\right\}$.
4. The set away from collision: $F_{1}\left(\epsilon \right)=F-\left(q_{di}\cup \partial F\cup F_{0}\left(\epsilon \right)\right)$.

The following theorem examines the properties of the proposed decentralized optimal navigation protocol via an overall multi-agent Navigation Function.

**Theorem 1.**  *Let $I_{1},I_{2}\subseteq R$ be intervals and let $\hat{\varphi }:F\to I_{1}$ and $\sigma :I_{1}\to I_{2}$ be analytic. Define the composition $\varphi :F\to I_{2}$ as $\varphi =\sigma \circ \hat{\varphi }$. If σ is monotonically increasing on $I_{1}$, then the set of critical points of $\hat{\varphi }$ and φ coincide and the (Morse) index of each critical point is identical.*

**Proof.**  The proof proceeds similarly to [19]. Since we have a connected workspace and a well-defined function $\gamma_{di}$, we first present some results for the navigation function $\varphi_{i}$ [20,26], which establish that the goal configurations are attainable without collisions and that there will always be a direction of movement decreasing the overall potential function. More specifically,

- Since the workspace is connected, the destination point $q_{di}$ is a non-degenerate local minimum of $\varphi_{i}$.
- All critical points of $\varphi_{i}$ are in the interior of the free space.
- For every ϵ>0, there exists a positive integer N(ϵ) such that if k>N(ϵ), then there are no critical points of ${\hat{\varphi }}_{i}$ in $F_{1}\left(\epsilon \right)$.
- There exists an $\epsilon_{0}>0$, such that ${\hat{\varphi }}_{i}$ has no local minimum in $F_{0}\left(\epsilon \right)$, as long as $\epsilon <\epsilon_{0}$.

Nevertheless, the aforementioned suppositions do not guarantee global convergence of the system state to the goal configurations on their own. To achieve such convergence, we define a time-invariant Lyapunov function for the overall system with respect to the positions of all agents. The candidate Lyapunov function is the sum of $\varphi_{i}$. Thus, differentiating the candidate Lyapunov function $\varphi =\sum_{i=1}^{m}\varphi_{i}$ along the trajectories of the multi-agent system (i.e., substituting the navigation protocol (7)), we obtain a negative semi-definite derivative. Thus, the value of the Lyapunov function decreases (i.e., no collisions occur) and moreover, the system state converges to the largest invariant set of the system, which is composed of the desired configuration and the rest of equilibria that, fortunately for sufficiently large parameters h,k, attain a measured zero domain of attraction. Therefore, the system trajectories converge safely to the desired configuration initializing from almost everywhere within the workspace. □

## 5. Results

**Figure 4:**
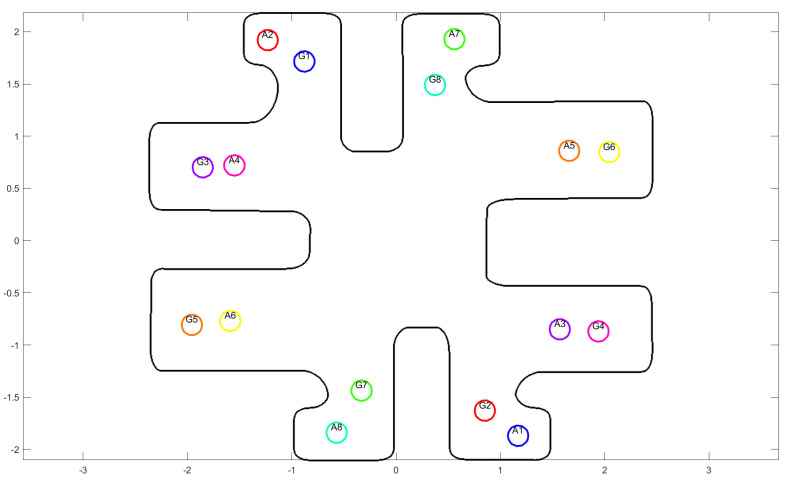
Workspace and the initial (symbol I) and desired (symbol G) position of each agent.

In this section, we will present the simulation results of the aforementioned method. We will use 8 mobile holonomic agents with radius r=0.1 which are in a workspace as shown in Figure 4 with the initial and desired positions given in Table 1. We will also run the same simulations with the Multi-Agent Poli-RRT* method to compare our method’s result. We will provide a short description of this comparison method in the next subsection. For the simulations, we use the programming and numeric computing platform MATLAB in version R2022b and a computer with a processor *Intel(R) Core(TM) i7-7500U CPU @ 2.70GHz 2.90 GHz* and RAM *8GB* (Intel, Santa Clara, CA, USA).

### 5.1. Multi-Agent Poli-RRT* Algorithm

The Multi-Agent Poli-RRT* method was first proposed by [40]. In that work, the authors extended the Poli-RRT* algorithm [47] to a non-holonomic Multi-Agent system in which the agents move in a workspace with internal obstacles by adopting a priority-based approach. That is, the agents are ranked according to a priority criterion and the algorithm finds trajectories in sequence, starting from the highest-priority agent and moving to the lowest one. Furthermore, in each iteration, when it is their turn, the agents with lower priority will learn about the trajectories of the previous higher-priority agents from an updated list. This list involves the edges of the agents’ trajectories and the time duration for which they were there. With this structure, the agents can prevent a possible collision and maintain a safe distance from the previous one. The aforementioned algorithm can be simply adjusted to our problem, and we adapt the agents’ controller to be similar to the (7).

### 5.2. Simulations

**Figure 5:**
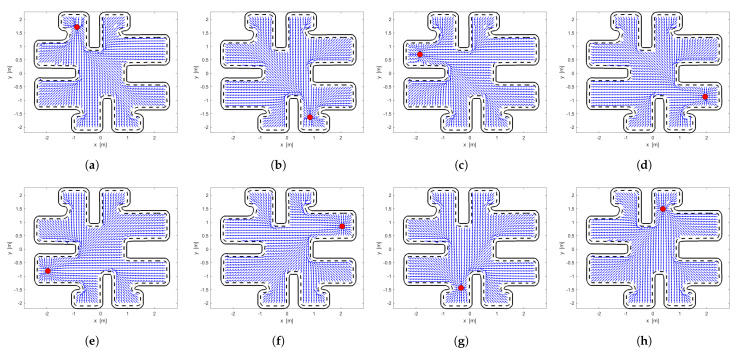
Optimal vector fields of each agent. The red-filled circle showcases the goal position. (**a**) Agent 1. (**b**) Agent 2. (**c**) Agent 3. (**d**) Agent 4. (**e**) Agent 5. (**f**) Agent 6. (**g**) Agent 7. (**h**) Agent 8.

Before we define the sub-optimal control law for each agent, we use the off-policy method to determine the optimal cost function for each agent. Because we want to attach equal importance to the minimization of the settling time and input response of the system (1), we set the w=0.5. In Figure 5, we illustrate the optimal vector fields of each agent using the method presented in [30]. Additionally, in Table 2 and Table 3 we present the time and cost value of each optimal policy, respectively.

As we mentioned earlier, to demonstrate the efficiency of the proposed sub-optimal controller, in this simulation, we examine how much the trajectory of each agent deviates from the optimal one of a SAS and the time that the global system will take to accomplish its task as we increase the number of agents starting simultaneously from the initial position. As the goal of the aforementioned MAS, we stated that all agents should reach their destination starting from the above initial position and following an optimal no-collision trajectory. To this end, we ran three simulations. In the first one, a group of two agents started towards their destination; in the second, a group of four started together; and in the last one, all the agents started together. We assume that an agent interacts only with the group’s members and does not affect the navigation of the other agents which are not in the group. Notably, using the same aforementioned properties, we implemented the comparison method, too. For each simulation, we chose the group of agents that would interact and the variables of sub-optimal control law ($k,\lambda ,k,\tau_{m}$).

For simulations, we categorized the agents into the following groups:

- Two members $\left(A_{1},A_{2}\right)$, $\left(A_{3},A_{4}\right)$, $\left(A_{5},A_{6}\right)$ and $\left(A_{7},A_{8}\right)$.
- Four members $\left(A_{1},A_{2},A_{7},A_{8}\right)$ and $\left(A_{3},A_{4},A_{5},A_{6}\right)$.
- Eight members $\left(A_{1},A_{2},A_{3},A_{4},A_{5},A_{6},A_{7},A_{8}\right)$ (all the agents).

For the Multi-Agent Poli-RRT* method, the first agent has the highest priority and the last one has the lowest.

As variables of sub-optimal control for each simulation, we define the values as follows:

- Simulation 1: $k=300,\lambda =NaN,h=NaN,\tau_{m}=2$. https://www.youtube.com/watch?v=-qLbfTVryj8 (accessed on 31 March 2024)
- Simulation 2: $k=250,\lambda =8,h=150,\tau_{m}=16$. https://www.youtube.com/watch?v=M2rhUSAz1w0 (accessed on 31 March 2024)
- Simulation 3: $k=200,\lambda =1,h=85,\tau_{m}=2$. https://www.youtube.com/watch?v=Z__lYbZY7O0 (accessed on 31 March 2024)

We have to notice that in simulation one, the variables λ=h=NaN because the complementary relations of each agent are not defined when we observe an interaction between two agents. Also, the selected variables are the best ones available, providing the best sub-optimal no-collision trajectory for each agent. Finally, the accompanying videos for each simulation are given in slow motion in order to observe the collision avoidance property.

**Figure 6:**
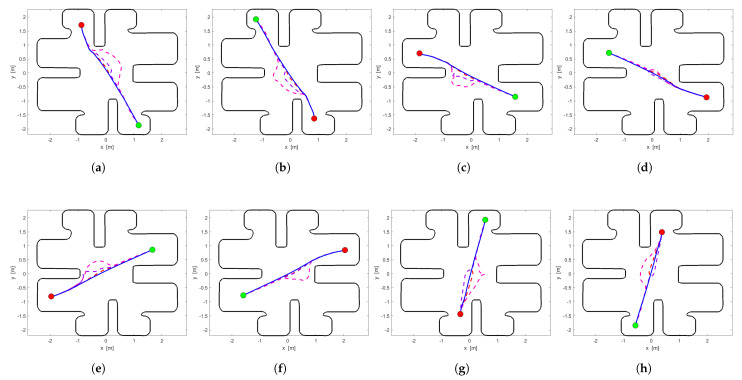
The Optimal (solid blue line), Simulation 1 (dashed red line), Simulation 2 (dashed purple line) and Simulation 3 (dashed magenta line) trajectories of each agent. The green-filled circle showcases the initial position and the red-filled circle showcases the goal position. (**a**) Agent 1. (**b**) Agent 2. (**c**) Agent 3. (**d**) Agent 4. (**e**) Agent 5. (**f**) Agent 6. (**g**) Agent 7. (**h**) Agent 8.

**Figure 7:**
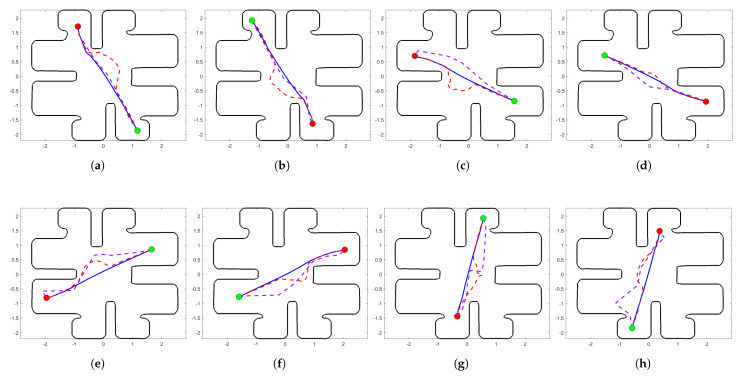
Agents’ optimal navigation (solid blue line) and their trajectories calculated with the sub-optimal navigation method (dashed red line) and Multi-Agents Poli-RRT* method (dashed purple line) at simulation 3. The green-filled circle showcases the initial position and the red-filled circle the goal position. (**a**) Agent 1. (**b**) Agent 2. (**c**) Agent 3. (**d**) Agent 4. (**e**) Agent 5. (**f**) Agent 6. (**g**) Agent 7. (**h**) Agent 8.

In Table 2, we list the time durations each agent needs to accomplish its tasks and in Table 3 we list the cost value of each agent for each simulation. Moreover, we also state the global system time duration and cost value in the last line. Both of the navigation methods’ results are shown in these tables too. Finally, in Figure 6 we have the optimal and sub-optimal greedy trajectory of each agent for optimal SAS and each simulation, and also in Figure 7 we present the trajectories of the two navigation methods during Simulation 3.

## 6. Discussion

In Figure 6, we provide the agents’ trajectories during the above simulations. Specifically, we see the optimal (solid blue line) and sub-optimal trajectories of each agent separately (dashed lines). In all simulations, at the start and the end of their trajectory, we notice that every agent is close to its optimal navigation. On the contrary, in the center of workspace, where the group of agents meets up, each agent deviates from its optimal trajectory or remains idle for a short time period in order to avoid conflicts with the other agents. As a result, the sub-optimal cost value increases as we upsize the members of the group. This occurs as a result of the tuning of the parameters of the sub-optimal controller according to Theorem 1, which is implemented to establish convergence from almost everywhere in the workspace. If we had not selected proper parameter variables, we would have watched the agents remain idle in the center of the workspace without accomplishing their tasks owing to the presence of a stable unwanted equilibrium.

In comparison with the Multi-Agent Poli-RRT* method, in Table 2 and Table 3, we showcase the global efficiency of our method. Also, we observe the efficiency of the priority attitude using the comparison method. That is, in Table 2 and Table 3, the highest-priority agents have a smaller time duration and cost value than the respective agents when our method is used. We observe the same result in Figure 7, which lists the trajectory results of the two methods during Simulation 3. The trajectories of the highest-priority agents (dashed purple line) are more limited in their optimal trajectory than sub-optimal (dashed red line). However, when the updated list increases in each iteration, the lowest-priority agents start to deviate from their optimal trajectory. For the above reasons, our approach is globally valid in comparison with the above priority criterion algorithm.

**Figure 8:**
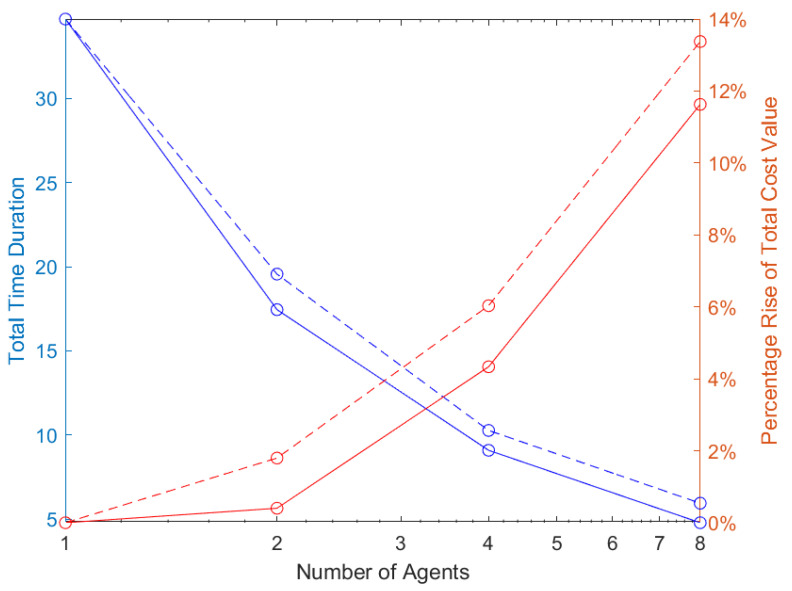
Total time duration of sub-optimal navigation method (solid blue line) and Multi-Agent PoliRRT* method (dashed blue line) and the percentage increase of the total cost value of the sub-optimal navigation method (solid red line) and Multi-Agent PoliRRT* method (dashed red line) according to the number of agents in the group.

Moreover, in the aforementioned results, as we expected, we observe a trade-off between total time duration and cost value. What we mean is that as we increase the number of agents that interact in the workspace, a decrease in the total time the agents require to accomplish their tasks occurs alongside an increase in the total cost value of MAS, since the agents deviate from their optimal trajectory. Figure 8 shows the decrease in the total time duration (blue lines) and percentage rise of the total cost value attached to the global optimal cost value (red lines) for the sub-optimal navigation (solid lines) and Multi-Agent Poli-RRT* (dashed lines) method where the horizontal axis is in logarithmic scale. This trade-off result provides additional confirmation of the global efficiency of our method.

## 7. Conclusions

In this paper, we propose a sub-optimal approach for the navigation problem of MAS in a simply connected workspace. We observed a trade-off between the time duration needed to fulfill the task and the overall cost value as we increase the number of members of a group. Moreover, we compare this method with the Multi-Agent Poli-RRT* to demonstrate the validity of our method compared to other research algorithms. We use this approach to avoid the computational complexity of RL-based methods, designing a sub-optimal decentralized control law. Despite the promising results of our study, there are several issues we should discuss. First of all, when using this method, the agents may be blocked by others if the workspace has narrow halls. Moreover, we cannot achieve global asymptotic navigation such as that referred to in the previous sections [19]. In future research, we will extend this methodology to ensure it remains functional in narrow workspaces, coordinating the agents properly to ensure they reach their destination, and we will utilize algorithms based on game theory to asymptotically approach the global optimal navigation of the system.

## Figures and Tables

**Table 1:** The initial and desired position of each agent.

Agent Index	Initial Position		Desired Position	
	x	y	x	y
1	1.17	−1.87	−0.88	1.72
2	−1.23	1.92	0.85	−1.63
3	1.57	−0.85	−1.85	0.70
4	−1.55	0.72	1.94	−0.85
5	1.66	0.86	−1.96	−0.81
6	−1.59	−0.77	2.04	0.85
7	0.56	1.93	−0.33	−1.44
8	−0.57	−1.84	0.37	1.49

**Table 2:** Time duration of each agent and whole system, with sub-optimal control method and Multi-Agent Poli-RRT* method.

Agents Index	Time Duration						
	Optimal SAS	Simulation 1		Simulation 2		Simulation 3	
		Sub-Optimal	Poli-RRT*	Sub-Optimal	Poli-RRT*	Sub-Optimal	Poli-RRT*
1	4.45	**4.45**	4.58	**4.55**	5.28	4.70	**4.52**
2	4.40	**4.45**	5.00	4.50	**4.43**	4.70	**4.52**
3	4.35	4.35	4.35	4.55	**4.33**	4.80	**4.76**
4	4.35	**4.35**	4.70	**4.45**	4.63	4.55	**4.48**
5	4.40	**4.40**	4.93	**4.55**	4.65	**4.60**	5.96
6	4.35	**4.40**	4.43	**4.40**	5.00	**4.70**	4.84
7	4.20	**4.25**	4.35	**4.30**	4.68	**4.50**	4.48
8	4.20	**4.25**	4.93	**4.35**	4.80	**4.35**	5.72
Total	34.70	**17.45**	19.56	**9.10**	10.28	**4.80**	5.96

**Table 3:** Cost value of each agent and whole system, with sub-optimal control method and Multi-Agent Poli-RRT* method.

Agents Index	Cost Value						
	Optimal SAS	Simulation 1		Simulation 2		Simulation 3	
		Sub-Optimal	Poli-RRT*	Sub-Optimal	Poli-RRT*	Sub-Optimal	Poli-RRT*
1	8.59	**8.60**	8.61	8.89	**8.65**	9.35	**8.64**
2	8.51	**8.53**	8.71	8.72	**8.63**	9.40	**8.67**
3	7.08	**7.11**	7.45	7.61	**7.09**	8.35	**7.40**
4	7.37	**7.40**	7.46	7.94	**7.56**	**8.33**	8.63
5	7.94	**7.97**	8.04	**8.30**	8.67	**8.63**	8.92
6	7.94	**7.97**	8.03	**8.05**	8.23	**9.14**	9.97
7	6.06	6.13	**6.10**	**6.26**	7.25	**6.85**	6.95
8	6.01	**6.05**	6.18	**6.32**	7.02	**6.38**	8.29
Total	59.51	**59.75**	60.58	**62.09**	63.10	**66.43**	67.47

## Data Availability

Data are contained within the article.

## Abbreviations

The following abbreviations are used in this manuscript:

MP	Motion Planning.
MAS	Multi-Agent System.
SAS	Single-Agent System.
CP	Centralized Policy.
DP	Decentralized Policy.
RRT	Rapidly-exploring Random Tree.
RL	Reinforcement Learning.
DRL	Deep Reinforcement Learning.
NF	Navigation Function.
AHPF	Artificial Harmonic Potential Field.
RPF	Relation Proximity Function.
RVF	Relation Verification Function.
